# Intestinal hypoperfusion in patients with Crohn's disease revealed by intraoperative indocyanine green fluorescence imaging

**DOI:** 10.1016/j.amsu.2021.102402

**Published:** 2021-05-24

**Authors:** Jun Higashijima, Toru Kono, Mitsuo Shimada, Hideya Kashihara, Chie Takasu, Masaaki Nishi, Takuya Tokunaga, Ayumu Sugitani, Kozo Yoshikawa

**Affiliations:** a Department of Digestive Surgery and Transplantation, Institute of Health Biosciences, The University of Tokushima, Kuramoto 3-18-15, Tokushima, 770-8503, Japan; b Advanced Surgery Center, Sapporo Higashi Tokushukai Hospital, 3-1, N-33, E-14, Higashi-ku, Sapporo, Hokkaido, 0650033, Japan; c Center for Clinical and Biomedical Research, Sapporo Higashi Tokushukai Hospital, 3-1, N-33, E-14, Higahi-ku, Sapporo, Hokkaido, 0650033, Japan

**Keywords:** Crohn's disease, Indocyanine green fluorescence imaging, Vascular perfusion

## Abstract

**Background:**

Anastomotic leakage has been reported as an independent risk factor for surgical recurrence at the anastomotic site in patients with Crohn's disease. An inadequate blood supply may contribute to this leakage. Real-time indocyanine green angiography has been useful for confirming vascular perfusion of the intestines. The aim of this study was to evaluate the use of intraoperative indocyanine green angiography to detect vascular perfusion of the intestines during ileocaecal resection in patients with Crohn's disease and colon cancer.

**Materials and methods:**

We retrospectively evaluated the medical records of 26 consecutive patients with colon cancer arising in the caecum or ascending colon and 3 consecutive patients with Crohn's disease without a history of disease-related surgery. The patients in the 2 cohorts had undergone ileocaecal resection at Tokushima University Hospital between January 2018 and January 2021. After ileocaecal resection, blood flow was evaluated in ileal (oral) and colon (anal) stapled stumps by indocyanine green fluorescence angiography. The fluorescence time was defined as the time from indocyanine green injection and flush of the injection route to the point when the stump showed the strongest fluorescent signal in the monitor.

**Results:**

The fluorescence time for the ileal and colon stumps in patients with Crohn's disease was 43.3 ± 8.8 s each and was significantly longer than the fluorescence time in the patients with colon cancer (29.4 ± 6.5 s and 29.6 ± 6.8 s, respectively) (P < 0.05).

**Conclusion:**

Intraoperative indocyanine green fluorescence imaging is safe and reproducible for assessing intestinal perfusion prior to anastomosis in patients with colon cancer and Crohn's disease.

## Highlights

• Low blood supply increases leakage and surgical recurrence in Crohn's disease.
• Indocyanine green angiography is useful for evaluating the intestinal blood supply.
• Blood supply at anastomotic sites was lower in Crohn's disease than colon cancer.

## 1 Introduction

Crohn's disease (CD)^1^ is a chronic, transmural, immune-mediated inflammation that can affect all layers of the intestine and results in considerable morbidity and diminished quality of life [1]. Surgical treatment for CD is a last option to improve quality of life, although the frequency of surgery has decreased because of the use of modern biological therapies and medical optimisation [1]. However, up to 70%–80% of patients affected by CD will require abdominal surgery in their lifetime, and up to 30% of these patients will require multiple surgeries because of disease recurrence [2,3].

A recent study of CD patients revealed that anastomotic leakage (AL) after bowel resection was an independent risk factor for surgical recurrence at the anastomotic site [4]. However, AL remains unpredictable. Inadequate blood supply at the anastomotic site may inevitably contribute to AL, although numerous risk factors are associated with this condition [5,6].

Real-time indocyanine green (ICG) angiography is a useful technology for confirming vascular perfusion of the intestines at the site of anastomosis [[7], [8], [9]]. Recently, we reported that the risk of AL after colorectal surgery could be objectively estimated by intraoperative ICG angiography [10]. The aim of the present study was to analyse our use of intraoperative ICG angiography to evaluate vascular perfusion of the intestines after ileocaecal resection (ICR) in patients with CD and colon cancer.

## 2 Materials and Methods

In this retrospective cohort study, we evaluated the medical records of patients who underwent laparoscopic ICR at the Tokushima University Hospital between January 2018 and January 2021. We examined the records of 26 consecutive patients who presented with colon cancer in the caecum or ascending colon and 3 consecutive patients with CD and no prior history of disease-related surgery. Exclusion criteria were patients with a history of adverse reaction to ICG and/or iodine, pregnant and/or lactating women, an age of ≥80 years, and individuals with clinical stage IV cancer. A high ligation of the ileocolic artery was defined as when the ileocolic artery travelled anterior and superior to the superior mesenteric vein in the ICR. A diagnosis of CD was based on conventional clinical, radiological, endoscopic, and histopathological criteria [11]. The study was approved by the institutional review board of Tokushima University Hospital (No. ToCMS 3215–2) and was performed in accordance with the ethical standards laid down in the Declaration of Helsinki. All included participants provided informed consent via public comments. This study was registered in the Research Registry (unique identifying number: researchregistry6770, https://www.researchregistry.com/browse-the-registry). This study has been reported in line with the STROCSS criteria [12].

During ICR, blood flow was evaluated in the ileal (oral) and colonic (anal) stumps using ICG (Diagnogreen; Daiichi Sankyo Co., Ltd., Tokyo, Japan) angiography before anastomosis. Intravenously injected ICG emits light with a peak wavelength of 800–850 nm. The ICG was visualised in the blood vessels after excitation with near-infrared light (760–780 nm) using the Hyper Eye Medical System (HEMS; Mizuho Medical Co., Ltd. Tokyo, Japan). Fluorescence time (FT) in both stumps was measured using the HEMS. The FT was defined as the time from ICG injection and flush of the injection route to the point where the strongest fluorescent signal was observed in the stump [10].

The graphs were created and calculations and statistical analyses were conducted using GraphPad Prism software, version 6.0, for Windows (GraphPad Software, San Diego, California, USA). To compare the four groups, one-way analysis of variance was performed. When there was a significant difference between groups, Tukey's multiple comparisons test was adopted for inter-group analysis. Data are presented as the mean ± standard deviation. The level of statistical significance was set at P < 0.05.

## 3 Results

The demographic details and characteristics of patients in the CD and colon cancer groups are presented in Table 1 and Table 2, respectively.

**Table 1:** Demographics and clinical characteristics of patients with Crohn's disease.

	Gender	Age, yr	Disease on-set	Disease location	Disease behavior	Type of anastomosis	Anastomotic leakage
Case 1	male	52	A3	L3	B2	Kono-S	No
Case 2	male	50	A3	L1	B2	Kono-S	No
Case 3	male	27	A1	L1	B2	Kono-S	No

**Table 2:** Demographics and clinical characteristics of patients with colon cancer.

Gender, male	n (%)	10 (38.5)
Age, yr	Median (range)	70 (43–76)
Pathological Stage		
0	n (%)	2 (7.7)
I	n (%)	9 (34.6)
II	n (%)	6 (23.1)
III	n (%)	9 (34.6)
Lymph node dissection		
D2	n (%)	12 (46.2)
D3	n (%)	14 (54.8)
High ligation of ileocolic artery resection	n (%)	14 (54.8)
Stapled side to side anastomosis	n (%)	26 (100)
Anastomotic leakage	n (%)	0 (0)

There were no intraoperative adverse events or conversion to open surgery. There were no adverse events related to the injection of ICG. ICG-enhanced fluorescence was detected in 100% of the patients. No changes in surgical plan occurred for any patient prior to ICG angiography. The type of anastomosis performed was the stapled side-to-side technique for patients with cancer or the Kono-S method for patients with CD. There was no AL in either group.

**Fig. 1:**
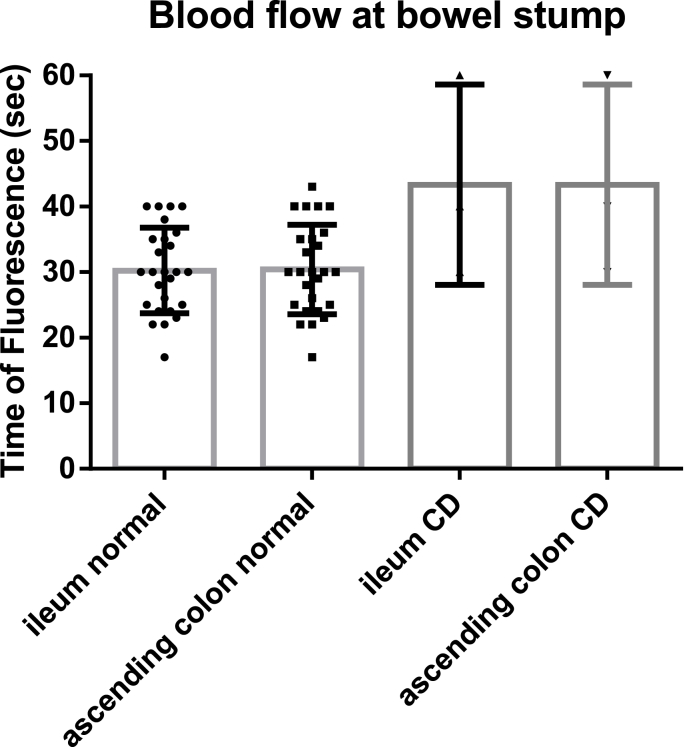
The fluorescence time (FT) of ileal and ascending colon stumps during ileocolic resection in CD and colon cancer patients. The FTs for both the ileal and ascending colon stumps in patients with CD was significantly longer than the FTs for the ileal and ascending colon stumps in the patients with colon cancer: ileum normal vs. ileum CD, P = 0.0007; ileum normal vs. ascending CD, P = 0.0006; ascending normal vs. ileum CD, P = 0.0035; and ascending normal vs ascending CD, P = 0.003. CD, Crohn's disease.

Fig. 1 shows the FTs for the ileal and ascending colon stumps were significantly longer in patients with CD than in patients in the colon cancer group (ileum normal vs. ileum CD, P = 0.0007; ileum normal vs. ascending CD, P = 0.0006; ascending normal vs. ileum CD, P = 0.0035; and ascending normal vs ascending CD, P = 0.003).

The FTs for the ileal and colonic stumps in patients with colon cancer were 29.4 ± 6.5 s and 29.6 ± 6.8 s, respectively (P = 0.918). The FT of the ileal and ascending colon stumps in patients with CD was 43.3 ± 8.8 s each. High ligation of the ileocolic artery did not affect the FTs of the ileal (28.8 ± 6.7 s for high ligation, 31.9 ± 6.2 s for low ligation) and ascending colon (29.1 ± 7.3 s for high ligation, 31.9 ± 6.2 s for low ligation) stumps in the colon cancer group (P = 0.2294 and P = 0.3112, respectively).

## 4 Discussion

We limited the surgical technique in our investigation to ICR to unify measurements in a specific region of the intestinal tract. This limitation minimised variability between the two study groups. The highlight of this study was the demonstration that vascular perfusion at the site of anastomosis was significantly lower in patients with CD compared with colon cancer patients.

Intraoperative judgement by the operating surgeon may be subjective and lead to underestimating the risk of AL based on visual inspection [13,14]. Therefore, various techniques have been developed for evaluating intestinal perfusion, such as ICG angiography and laser Doppler flowmetry [[15], [16], [17]]. Comparisons between ICG fluorescence imaging, intraoperative Doppler ultrasound, laser Doppler flowmetry, and oxygen spectroscopy have not been widely attempted because these techniques cannot be easily applied during surgery. However, intraoperative ICG fluorescence imaging has been shown to be a potential tool to assess bowel perfusion and reduce the risk of AL in colorectal surgery for cancer [7,9].

CD is a chronic, transmural inflammatory process that also damages the intrinsic intestinal nervous system. The submucosal plexus is particularly susceptible to damage because of its location and morphology. Regeneration of nerve fibres may take years compared with weeks for mucosal healing. Therefore, even after mucosal healing is complete, neuronal regeneration may be incomplete.

It has been reported that regional total intestinal blood flow in patients with CD was reduced by 50% compared with healthy individuals, and this result may have been due, in part, to reduced levels of the potent vasodilator calcitonin gene-related peptide in the submucosal plexus [18,19]. A decrease in this peptide may be a partial explanation for the decreased intestinal blood flow observed in the present study for patients with CD when compared to colon cancer patients. This possibility may be supported by evidence that suggested the ileocolic AL rate in patients with CD increased up to 14.3% [20]. Thus, intraoperative ICG fluorescence imaging appears to aid in identifying issues with intestinal blood flow and may be used to implement changes in surgical plans for CD patients at high-risk for AL.

The major limitation of our retrospective study was the small sample size. However, intraoperative ICG fluorescence imaging appears to be safe and reproducible for assessing intestinal perfusion before anastomosis in patients with CD and colon cancer.

## 5 Conclusions

Intraoperative indocyanine green fluorescence imaging is safe and reproducible for assessing intestinal perfusion prior to anastomosis in patients with colon cancer and CD. The FT for perfusion in the ileal and colon stumps in CD patients was significantly longer than that in patients with colon cancer. Future research with larger numbers of patients will be required to further evaluate the usefulness of ICG angiography during surgical interventions for CD.

## Funding

This work was not supported by any funding agencies.

## Declaration of interest

None.

## Author contributions

All authors contributed to the design of the study and approved the final version of the manuscript. JH, TY, SE, HK, CT, MN, AS and TT contributed to acquisition of the data. JH, TK, MS, and KY contributed to interpretation of the data. TK and MS drafted the article.

## Data availability statement

The data underlying this article will be shared on reasonable request to the corresponding author.

## Provenance and peer review

Not commissioned, externally peer-reviewed.

## Footnotes

1 **Abbreviations:** AL, anastomotic leakage; CD, Crohn's disease; FT, Fluorescence time; ICG, indocyanine green; ICR, ileocaecal resection.
